# Phenotypic manifestation of congenital transverse amputation of autopod in Pakistani subjects

**DOI:** 10.12669/pjms.322.8850

**Published:** 2016

**Authors:** Hafiza Fizzah Riaz, Karmoon Lal, Saif Ullah, Nadeem Ahmad Bhatti, Waheed Ullah, Sajid Malik

**Affiliations:** 1 Hafiza Fizzah Riaz, Human Genetics Program, Department of Animal Sciences, Faculty of Biological Sciences, Quaid-i-Azam University, 45320 Islamabad, Pakistan; 2 Karmoon Lal, Human Genetics Program, Department of Animal Sciences, Faculty of Biological Sciences, Quaid-i-Azam University, 45320 Islamabad, Pakistan; 3 Saif Ullah, Human Genetics Program, Department of Animal Sciences, Faculty of Biological Sciences, Quaid-i-Azam University, 45320 Islamabad, Pakistan; 4 Nadeem Ahmad Bhatti, Human Genetics Program, Department of Animal Sciences, Faculty of Biological Sciences, Quaid-i-Azam University, 45320 Islamabad, Pakistan; 5 Waheed Ullah, Human Genetics Program, Department of Animal Sciences, Faculty of Biological Sciences, Quaid-i-Azam University, 45320 Islamabad, Pakistan; 6 Sajid Malik, Human Genetics Program, Department of Animal Sciences, Faculty of Biological Sciences, Quaid-i-Azam University, 45320 Islamabad, Pakistan

**Keywords:** Limb amputations, transverse defects, terminal deficiency, symbrachydactyly, monodactyly, Pakistani subject

## Abstract

Terminal transverse deficiency of forearm is a very rare limb malformation. Most of the cases have traumatic etiology and congenital presentation is less common. A series of six individuals with transverse deficiency through the hands is presented in this communication. The cases were congenital, morphologically similar and showed loss of four fingers, most often postaxial. The affected arm was reduced in size compared to the contralateral limb and there was distortion of palmer creases. All cases were sporadic and non-syndromic in nature. The characteristics of these cases were concordant with the symbrachydactyly type III or monodactylous type, when classified according to the scheme proposed by Blauth and Gekeler (1973). The malformation resulted in permanent quality-of-life impairment in these subjects and warrant prosthetic management. Detailed physical and phenotypic features of the patients have been presented.

## INTRODUCTION

Terminal transverse amputations are characterized by the absence of distal portions of the extremities extending across the width of the limb/autopod.^1^, ^2^ Hand amputation most often has traumatic etiology and its congenital presentation is very rare. In most of the reported cases it had unilateral, isolated and sporadic occurrence.^3^, ^4^

Data on congenital transverse amputation (CTA) in Pakistani patients are scarce and there is no systematic documentation of such cases. In this communication, we present six individuals with variable degrees of CTA through hand/palm.

## SUBJECTS

Six individuals (4M,2F) with CTA were ascertained during 2011-2015 from different regions across Pakistan (Table-I). Clinical evaluations were performed with the help of local physicians and the cases were classified according to the scheme proposed by Blauth and Gekeler.^5^ An informed consent was obtained from each individual or his/her parents. The study was approved by the Ethical Review Committee of the Quaid-i-Azam University, Islamabad.

**Table-I:** Demographic attributes and phenotypic manifestation in individuals with hand/palm amputation.

Variable	Individual					

	I	II	III	VI	V	VI
*Demographics*						
Gender/age(yrs)	M/7	M/20	F/8	F/16	M/12	M/8
Geographic origin	Southern-Punjab	Interior-Sindh	Upper-Punjab	South-KPK	North-KPK	North-KPK
Caste/language	Arain/Punjabi	Lashari/Saraiki	Laang/Saraiki	Pathan/Pushto	Khowar/Shauteye	Swati/Pushto
Parental consanguinity	Distantly related	First cousin	Distantly related	Non-related	Non-related	Non-related
Paternal and maternal age at patient’s birth (year)	40/38	20/18	29/22	27/23	29/28	37/30
Patient’s parity	4 of 4	1 of 5	1 of 3	1 of 7	3 of 4	7 of 9
No. of normal sibs (B:S)	0:1	1:3	1:1	4:2	1:2	3:5
*Phenotype*						
Affected hand	Left	Right	Right	Left	Left	Left
Amputation axis	Palm, median	Palm, proximal	Palm, median	Palm, median	Palm, proximal	Phalanges, proximal
Fingers	Bead like remnants of fingers 2-5	Digits 2-5 absent	Bead like remnants of fingers 2-5;	Digits 2-5 absent	Digits 1-4 absent, 5^th^ digit present	All fingers affected
Thumb	Terminal hypoplasia, short nail	Short, distal symphlangism	Terminal hypoplasia	Unaffected	Absent	Terminal hypoplasia, short nail
Affected arm, reduced/short	+ +	+	++	+	++	No
Contralateral arm	Mild shortening of zeugopod and stylopod	Medial inclination of index finger; crowding of carpals	Left thumb with extra palmer creases	Unaffected	Unaffected	Unaffected
Others	Carpals absent; hypoplastic metacarpals	Fused carpals; metacarpals 2-4 not visible; reduced metacarpal 5	Swelling on left throat			

+=mild; ++=moderate.

The CTA of hand was essentially unilateral in all the cases and there was no family history of limb or any other congenital malformation. There were amputations of variable degrees which resulted in loss of four fingers, mostly postaxial (Table-I). The individuals also exhibited affected limb-length discrepancy compared to the contralateral arm. Palmer creases were distorted. In five of the six cases the feet were unremarkable. All individuals had normal IQ and no associated anomaly was evident in gross physical examination. The patients had functional restrictions in their daily/occupational lives. The snapshot of CTA phenotype is given below:

### Case I

**Fig.1:**
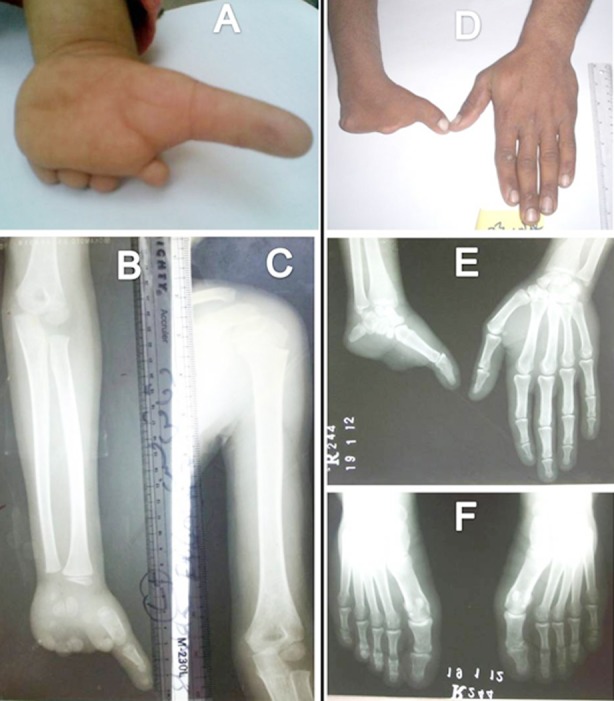
(A-C): Photographs and roentgenographs of individual I. (D-F): Phenotype in individual II.

The individual was fourth in the sibship of four. Reportedly, the first pregnancy of his mother was delayed for three years and the first two sibs (females) died in postpartum. The individual was observed to have CTA through his left palm. Fingers 2-5 were represented by bead-like remnants and there was distortion of palmer dermatoglyphics (Fig.1A). Roentgenographs revealed aplastic/hypoplastic carpals, absent metacarpals, terminal hypoplasia of first digital ray, and mild shortening of radius/ulna (Fig.1B-C).

### Case II

This male patient was observed to have CTA of right hand through the palm. A short thumb was evident in the affected hand (Fig.1D). Roentgenographs depicted absence of several carpals and metacarpals; metacarpal 5 was represented by a small peg-like osseous element, and there was terminal symphalangism of first digital ray (Fig.1E). In the left hand, there was medial inclination of index finger and crowding of carpals. In the feet, there was bilateral hypertrophy of first digital ray with hallux valgus (Fig.1F).

### Case III

**Fig.2:**
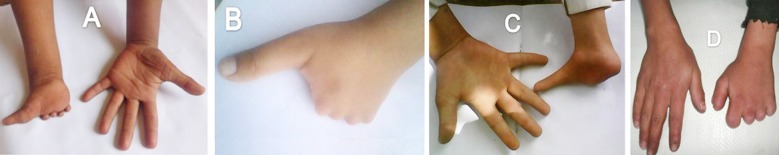
(A): Amputation in individual III; (B): individual IV; (C): individual V. (D); individual VI.

There was CTA of right hand which culminated in four nubbin-like digits and a relatively normal thumb (Fig.2A). Characteristic dermatoglyphics were evident.

### Case IV

The individual had CTA through the medial axis of left palm and digits 2-5 were completely omitted. The thumb appeared unaffected (Fig.2B).

### Case V

The left hand exhibited CTA through the proximal plan of palm (Fig.2C). Fingers 1-4 were completely omitted and only the 5^th^ finger was present. The affected arm was markedly reduced in size.

### Case VI

The individual had CTA through the left hand (Fig.2D). Fingers 2-5 were amputated at their bases while the thumb showed terminal deficiency.

## DISCUSSION

The CTA is generally reported as symbrachydactyly. In a retrospective study on patients with terminal amputations, Kallemeier et al.^3^ concluded that transverse deficiency through the forearm represents a proximal continuum of symbrachydactyly. The six cases presented here show remarkable similarities with each other and were concordant with symbrachydactyly type III or monodactylous type;^5^ the hallmark of this type is the absence of all fingers other than the thumb, including parts of the metacarpals. Interestingly, in one of our cases the amputated fingers were preaxial including the thumb and only the 5^th^ finger was present.

Nubbin-like digits appear to be an occasional feature of symbrachydactyly. Two of our patients also exhibited soft nubbin-like finger remnants at the distal border of affected hand. Kallemeier et al.^3^ observed that 71% of the 291 patients with upper-extremity transverse deficiency had soft tissue nubbins at the end of their amputations. We also observed that left hand was more commonly affected than the right. De Smet et al.^4^ recruited patients with symbrachydactyly and observed that the involvement of left hand was twice as common as right. The authors also witnessed that unilateral cases were customary.

The unilateral and isolated nature in most of the cases of symbrachydactyly supports the nongenetic etiology. However, involvement of this anomaly with other syndromes likes Adams-Oliver syndrome (OMIM-100300) and ADAM complex (OMIM-217100) may suggest genetic factors in certain types. Other hypothesis than the vascular disruption explaining symbrachydactyly and associated syndromes have been proposed.^6^ The mesenchymal failure of the arm bud may cause terminal aplasia or intercalated deficiency.

Hand amputations have devastating effect on the lives of the individuals. CTA have not gained much attention in Pakistan.^7^, ^8^ CTA not only affect the functioning of hand but also put great social and psychological burden on the patients.^8^, ^9^ Such individuals remain highly disadvantaged in daily and occupational lives. There is a dire need to launch further studies for a comprehensive understanding of the prevalence, determinants and etiology of these anomalies in Pakistan.
